# Leprosy trends at a tertiary care hospital in Mumbai, India, from 2008 to 2015

**DOI:** 10.3402/gha.v9.32962

**Published:** 2016-11-23

**Authors:** Thirumugam Muthuvel, Petros Isaakidis, Hemant Deepak Shewade, Lucy Kattuppara, Rajbir Singh, Srinivas Govindarajulu

**Affiliations:** 1 German Leprosy and TB Relief Association, Chennai, India; 2 Médecins Sans Frontières (MSF)/Doctors Without Borders, Mumbai, India; 3 The Union, South East Asia Office, New Delhi, India; 4 Vimala Dermatological Centre, Mumbai, India

**Keywords:** leprosy, child cases, reconstructive surgery, migrants

## Abstract

**Background:**

Leprosy remains an important cause of preventable disabilities. After the advent of multidrug therapy, new leprosy cases have come down dramatically. Despite this achievement, India, which contributes 60% of the global leprosy burden, faces some challenges to eliminate the disease, including active transmission in the community and delayed diagnosis of leprosy patients.

**Objectives:**

The objectives of the study were 1) to determine sociodemographic and clinical characteristics of newly diagnosed adults and children (less than 15 years) with leprosy and their trends over time (2008–2015) and 2) to describe the profile of surgical procedures among leprosy patients registered for reconstructive surgeries during 2006–2015.

**Design:**

Retrospective descriptive study was conducted involving a record review of new patients with leprosy registered in Vimala Dermatological Centre, Mumbai.

**Results:**

A total of 578 new leprosy cases were registered in the hospital during 2008–2015. There has been a steady increase in the trend of child cases (less than 15 years) registered in the facility (from 3% in 2008 to 18% in 2015), *x*^*2*^=12.11, *p<*0.01. The majority of the patients (68%) were migrants of Uttar Pradesh and Bihar.

**Conclusions:**

Targeting children and migrants and ensuring early diagnosis and treatment initiation are essential components for leprosy elimination in an urban metropolis in India.

## Introduction

India contributes 60% of the global burden of leprosy (1). On January 30, 2006, the Government of India announced the ‘elimination of leprosy as a public health problem at the national level’ (2). Unexpectedly, this term of elimination in leprosy has been a misnomer and has been misunderstood as eradication. This has somehow allowed low priority of leprosy among funders, health planers, and health care providers and even among the community. Elimination was declared when the prevalence rate reduced to less than 1 case per 10,000 population at the national level. It was actually an achievement of intensified control (something between control and elimination of disease) and not ‘elimination or eradication of leprosy as disease’ (zero incidence of leprosy) or eradication of leprosy infection (3). In the same year, the Government of India instructed to ‘stop all active search for case detection’ and integrate the management of the diseases into the general health system (4). Since then, efforts to detect cases have significantly slowed down. A total of 125,785 new cases were detected during 2014–2015 in India of which 9% were child cases, indicating active transmission of leprosy in Indian communities. In Maharashtra, 12.5% were reported to be children among the new leprosy patients during the same period (1).

Charitable hospitals have played a vital role all through the history of leprosy control in India. One of the major tasks undertaken by them is running referral care centers and even in the post-integration phase, specialized services offered by such hospitals remain relevant (5). Understanding the profile of new leprosy cases in referral care centers, especially after the year of integration of leprosy in the general health system, is important. The study of trends of new cases and reconstructive surgeries (RCSs) over time may help define strategies in the leprosy program as well offer insights into the needs for resource allocation. The aim of this study was to describe the epidemiological and clinical profile of leprosy patients in a tertiary care referral hospital in Mumbai, India. Specific objectives were: 1) to determine sociodemographic and clinical characteristics of newly diagnosed leprosy cases among adults and children (less than 15 years) and study their trends over time (2008–2015; and 2) to describe the profile of surgical procedures among leprosy patients registered for RCS during 2006–2015.

## Methods

### Study design

Retrospective descriptive study was conducted involving record review.

### Setting

#### General setting

Mumbai is a metropolitan city, one of the most populous cities in India, with high migration throughout the year. Population of Mumbai in 2011 was estimated to be 12,440,000 (6). During 2010–2011, number of new cases reported in Mumbai was 792. Currently (2014–2015) the new cases reported was 565 and the annual new case detection rate was 2/100,000 population.

#### Study setting

Vimala Dermatological Centre (VDC), a charitable tertiary care referral facility located in Mumbai, has been providing care and treatment for leprosy since 1976. Work in the center includes raising awareness and providing medical treatment in the hospital. VDC is recognized as one of the RCS center by the Government of India and supported by the German Leprosy and TB Relief Association.

### Study population

For Objective 1 all new leprosy patients registered in VDC (2008–2015) and for Objective 2 all leprosy patients who underwent RCS (2006–2015) were included in the study.

### Data variables, sources of data, and data collection

Data were collected routinely through paper-based treatment cards. Consolidated reports were entered in a Microsoft Excel database every month. All paper-based records/registers were stored in health facility and electronic records were stored in a separate computer.

We extracted data from the electronic database. For Objective 1, the following variables were collected during registration: year, age, gender, duration of stay in Mumbai before registration, state of domicile, type of leprosy (pauci or multibacillary [MB]), and disability (Grade 0/1/2). For Objective 2, the following variables were collected: RCS type (hand/foot/eye), age, sex, and year of surgery.

### Analysis and statistics

Data analysis was carried out in Microsoft Excel and online statistics calculator (http://epitools.ausvet.com.au/content.php?page=trend). Mean and standard deviation were used to summarize continuous variables. Frequency and proportion were used to summarize categorical variables. Trend analysis was carried out based on the distribution; moving averages were established for number of new cases registered and chi-square test for linear trend was established for proportion of child cases and Grade 2 disability.

### Ethics approval

Ethics approval was obtained from Ethics Advisory Group of the International Union Against Tuberculosis and Lung Disease (The Union), Paris, France. As this study involved review of existing records, waiver of written informed consent was sought and approved. Administrative approval was obtained from the VDC hospital before the start of the study.

## Results

A total of 578 new leprosy cases were registered in the hospital during 2008–2015. The sociodemographic characteristics of the new cases registered are summarized in Table 1. In 2008 the average age of a new case registered in the facility was 32 years, while in 2015 it was 26 years. Ten percent of the new patients were children less than 15 years of age. Twenty-seven percent of the new patients registered in 2008 were women and girl children, and in 2015, the proportion was 36%. There was a male preponderance with M: F ratio of 2.3: 1.

**Table 1:** Sociodemographic characteristics of newly registered leprosy patients in a tertiary leprosy center, Mumbai, 2008–2015

Year	Total new cases (*n*)	Mean age (years)±SD	Females, *n* (%)	Child, *n* (%)	Duration of stay in Mumbai (years), median (IQR)
2008	60	31.5±13.8	16 (27)	2 (3)	8.5 (1–18.8)
2009	80	30.8±14.6	20 (25)	5 (6)	10 (2–18)
2010	73	31.5±16.5	26 (36)	4 (5)	10 (2–16.5)
2011	50	29.1±12.3	11 (22)	4 (8)	5 (2–18.5)
2012	90	30.2±13.6	24 (27)	9 (10)	10 (2–20)
2013	76	31.6±15.8	25 (33)	6 (8)	10 (2–16.5)
2014	75	31.3±16.4	26 (35)	12 (16)	9 (2–20)
2015	74	25.6±12.7	27 (36)	13 (18)	7.5 (2–16)
Total	578	30.2±14.7	175 (30)	55 (10)	10 (2–17)

IQR, interquartile range; SD, standard deviation.

Table 2 displays the proportion of MB type, Grade 2 disability among new cases. Nine percent had leprosy reaction at initial presentation to the hospital. Proportion of Grade 2 cases among new cases was 2% in 2008 and 11% in 2015.

**Table 2:** Clinical characteristics among newly registered leprosy patients in a tertiary leprosy center, Mumbai 2008–2015

	Total number of new patients registered	MB cases	Grade 1 cases	Grade 2 cases
	
Year	*N*	*n* (%)	*n* (%)	*n* (%)
2008	60	22 (37)	3 (5)	1 (2)
2009	80	34 (43)	3 (4)	5 (6)
2010	73	28 (38)	6 (8)	9 (12)
2011	50	23 (46)	7 (14)	2 (4)
2012	90	52 (58)	23 (26)	14 (16)
2013	76	46 (61)	15 (20)	5 (7)
2014	75	35 (47)	15 (20)	10 (13)
2015	74	34 (46)	9 (12)	8 (11)
Total	578	274 (47)	81 (14)	54 (9)

MB, multibacillary; VDC, Vimala Dermatological Centre.

Table 3 displays the type of RCS in the hospital. A total of 253 RCSs were performed during 2006–2015. Majority (58%) of the surgeries performed in the facility were hand surgeries for claw hand and foot surgeries (38%) for foot drop.

**Table 3:** Year-wise performance of reconstructive surgery in VDC, Mumbai, during 2006–2015

	Registered for RCS	Hand surgeries	Foot surgeries	Eye surgeries	Child cases
	
Year	*N*	*n* (%)	*n* (%)	*n* (%)	*n* (%)
2006	25	13 (52)	12 (48)	0 (0)	0 (0)
2007	41	27 (66)	11 (27)	3 (7)	8 (20)
2008	35	16 (46)	18 (51)	1 (3)	3 (9)
2009	32	19 (59)	13 (41)	0 (0)	1 (3)
2010	34	17 (50)	15 (44)	2 (6)	3 (9)
2011	26	10 (38)	15 (58)	1 (4)	5 (19)
2012	14	6 (43)	5 (36)	3 (21)	0 (0)
2013	15	14 (93)	1 (7)	0 (0)	4 (27)
2014	15	12 (80)	3 (20)	0 (0)	1 (7)
2015	16	14 (88)	2 (13)	0 (0)	0 (0)
Total	253	148 (58)	95 (38)	10 (4)	25 (10)

RCS, reconstructive surgery; VDC, Vimala Dermatological Centre.

The majority of the patients registered in the facility were migrants (68%, *n=*390), mainly from Uttar Pradesh (38%, *n=*217) and Bihar (8%, *n=*46) (Supplementary Table 1).

**Fig. 1:**
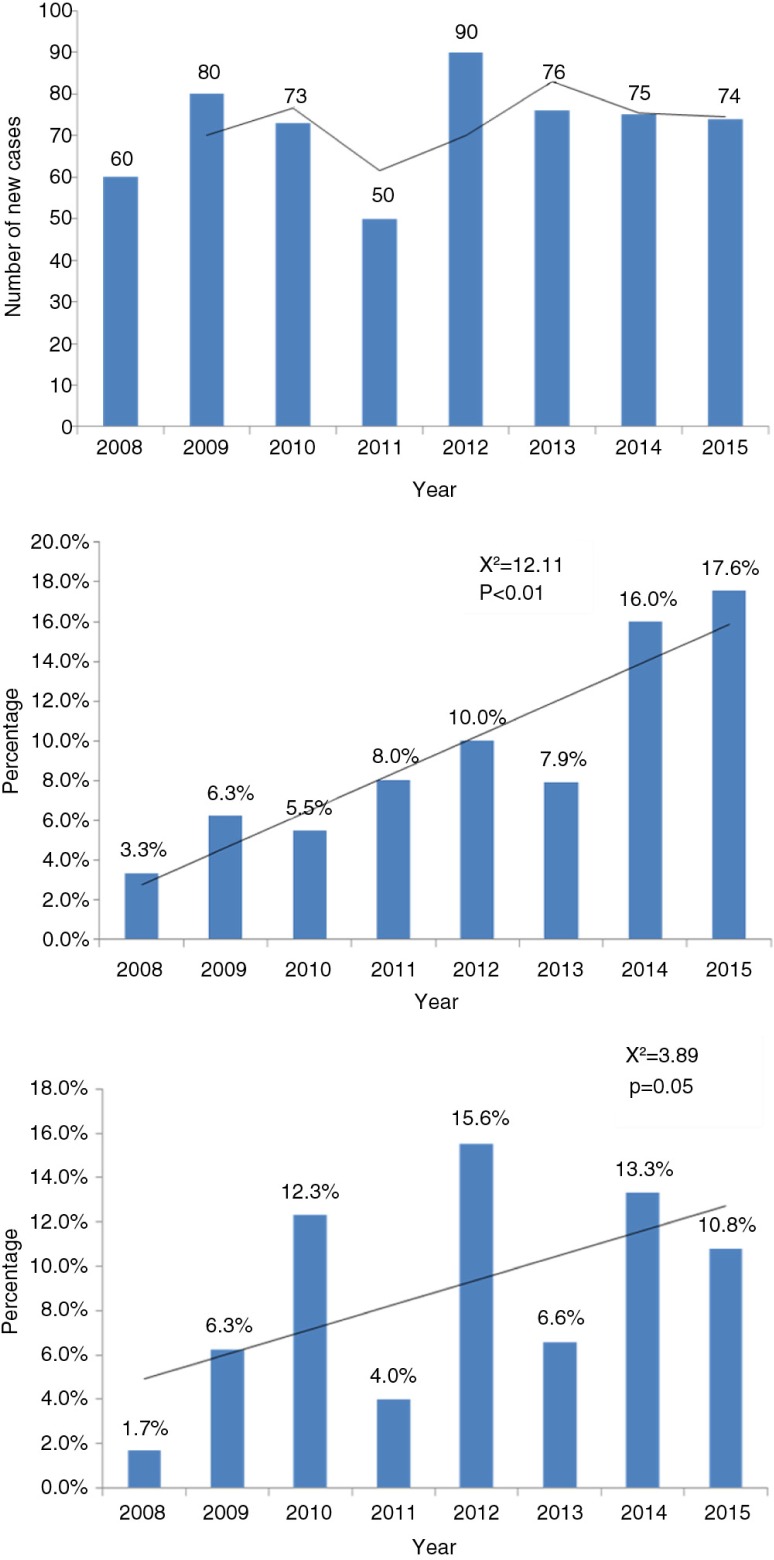
New leprosy patients registered, and proportion of child cases and Grade 2 disability among the new cases during 2008–2015 in VDC, Mumbai, and trend overtime.

Figure 1 displays number of new leprosy patients registered, proportion of child cases, and proportion of Grade 2 disability among new cases during 2008–2015 in VDC, Mumbai, and trend overtime.

There has been a steady increase in the trend of child cases registered in the facility (from 3% in 2008 to 18% in 2015) (*x*^*2*^=12.11, *p*<0.01). There has been a fluctuating trend in the proportion of new cases with Grade 2 disability (*x*^*2*^=3.89, *p*=0.05; Fig. 1).

## Discussion

This study from Mumbai revealed that there was an increase in the proportion of children among the newly registered patients with leprosy at a tertiary center in Mumbai. This indicates active transmission of leprosy in the community, despite important achievements in the fight against leprosy in India and the world. A large proportion of migrants were among the newly registered patients; hand surgery was the most frequently performed reconstructive surgical procedure.

The majority of the new leprosy patients registered in the facility belonged to middle age group, similar to a finding by Chhabra et al. and Jindal et al. (7, 8). The higher male to female ratio (2.3:1) in our study is also similar to the study by Chhabra et al. Mostly men migrate to cities (38% from Uttar Pradesh and 33% from Maharashtra) for search of better employment, and this could partly explain the higher proportion of males in our study (7).

Maharashtra reported 12.5% new child cases in 2015 while the national average during the same period was 9%. In this study, the child leprosy cases reported during 2015 was 18% which is higher than the state average as well as the national average. The overall proportion of child cases reported was 10% in the last 8 years. A study in Akola district of Maharashtra reported an increase in proportion of child cases from 8% in 2010 to 34% in 2014 (9). The study by Palit et al. reported occurrence of childhood leprosy in tertiary care hospitals varied from 5.1 to 11.4% (10). The study by Singal et al. in a tertiary hospital in Delhi (metropolitan city) reported 9.6% child cases and 12.8% Grade 2 deformity cases during (2000–2009). This finding is comparable with the proportion of child cases reported in our study (11). Our data add to the evidence that leprosy likely continues to be transmitted in the communities.

The percentage of MB cases was 47% in our study, slightly lower than the state average of 53% and the national average of 65%. A study from Satara district of Maharashtra in 2007–2008 reported 54% MB cases (12). The proportion of Grade 2 cases and Grade 1 cases among the new patients was 9 and 14% respectively. This proportion is slightly higher than the state average of 5.8% (Grade 2) and 4.3% (Grade 1) and the national average of 4.6% (Grade 2) and 5.2% (Grade 1) (13). This increase in proportion of Grade 2 and Grade 1 cases could be due to referral from various centers to this tertiary hospital. One of the stated strategies in enhanced Global Leprosy Strategy (2011–2015) to reduce deformities was to improve early detection of leprosy reaction as well as the role of careful neurological examination (14).

A total of 16 RCSs conducted in 2015, and there has been fluctuating trend in the number of RCSs conducted in the facility over the years. There were few facilities with expertise in conducting RCSs in overtime, and this partly explain the fluctuating trend of RCSs performed in the facilities. The proportion of hand RCS had increased to 88% in 2015, indicating that functional usage–related RCSs has increased in the recent years.

The proportion of migrants was high in the study group. Mumbai being a major urban area, it poses unique challenges for health services management. The challenges include inequalities due to the social, cultural, and economic context making vulnerable segments unable to access health services.

This was a retrospective data analysis based on departmental records; hence, bias in reporting cannot be ruled out and one should be careful of generalisation of results. Since the health facility is a tertiary care facility, we could include only the cases presenting to the center and we can assume more Grade 2 disability complicated cases were being recorded. Subgroup analysis on treatment compliance in migrant population is not presented and it is a limitation of the study.

The health facility registered 13% of the district's new leprosy burden in 2015. This health facility being among the few specialized centers and leprosy not being diagnosed and treated widely, the study population is a representative of the general population. Hence, the findings will be applicable for the district of Mumbai. In conclusion, the data from a tertiary referral center suggest that the proportion of new cases is slightly increasing, especially among children, compared with the national average, and this needs further study. The proportion of Grade 2 and Grade 1 cases is also more than the state and national average, indicating a late presentation and delayed diagnosis. Increase in new leprosy cases in children, MB-type leprosy, leprosy in women, and new cases with Grade 2/1 deformity are some indications for active transmission of leprosy in the community. Targeted intervention should focus in the States‘/Districts’ of high leprosy endemicity. The indigenous cases are pretty high too in our study. Focusing on children and migrant population and enforcing awareness and early diagnosis and treatment initiation are essential measure to eliminate leprosy in a metropolitan urban area like Mumbai.

## Supplementary Material

Leprosy trends at a tertiary care hospital in Mumbai, India, from 2008 to 2015

## Paper context

India, which contributes 60% of the global leprosy burden, has declared elimination of leprosy as a public health problem in 2006 and integrated leprosy in general health services. New leprosy cases however are reported every year. A retrospective record review conducted in a tertiary hospital in Mumbai revealed an increasing number of new pediatric cases in the past 8 years, indicating active transmission in the population and highlighting the urgent need for community interventions.
