# Supplementary material for: Leprosy trends at a tertiary care hospital in Mumbai, India, from 2008 to 2015
Source: Glob Health Action. 2016 Nov 23;9:10.3402/gha.v9.32962. doi: 10.3402/gha.v9.32962 (PMC5123210; doi:10.3402/gha.v9.32962)
Supplement: Leprosy trends at a tertiary care hospital in Mumbai, India, from 2008 to 2015 [file GHA-9-32962-s001.pdf]

Supplement 1: Residency status of the study population

State/Country	Number of cases	%
Uttar Pradesh	217	37.5
Maharashtra	188	32.5
Bihar	46	8.0
Karnataka	27	4.7
Andhra Pradesh	17	2.9
Gujarat	15	2.6
**Nepal**	**15**	**2.6**
Madhya Pradesh	10	1.7
Tamil Nadu	10	1.7
Odisha	9	1.6
Rajasthan	5	.9
West Bengal	5	.9
Others	14	2.0
